# Investigating the Role of FABP4 in Diabetes and Obesity and the Influence of Age and Ethnicity: A Comprehensive Analysis of a Cohort from the KEDP-Study

**DOI:** 10.3390/ijms25094578

**Published:** 2024-04-23

**Authors:** Mohammed A. Abdalla, Jehad Abubaker, Mohamed Abu-Farha, Irina Al-Khairi, Preethi Cherian, Mohammad G. Qaddoumi, Fatema Al-Rashed, Thangavel Alphonse Thanaraj, Ahmed N. Albatineh, Fahd Al-Mulla

**Affiliations:** 1 Department of Translational Research, Dasman Diabetes Institute, Kuwait City 15462, Kuwait; mohammed.ahmed@dasmaninstitute.org (M.A.A.); mohamed.abufarha@dasmaninstitute.org (M.A.-F.); 2 Hull York Medical School (HYMS), University of Hull, Hull HU6 7RX, UK; 3 Department of Biochemistry and Molecular Biology, Dasman Diabetes Institute, Dasman 15462, Kuwait; jehad.abubakr@dasmaninstitute.org (J.A.); irina.alkhairi@dasmaninstitute.org (I.A.-K.); preethi.cherian@dasmaninstitute.org (P.C.); 4 Pharmacology and Therapeutics Department, Faculty of Pharmacy, Kuwait University, Kuwait City 13110, Kuwait; mohammad.qaddoumi@dasmaninstitute.org; 5 Department of Immunology & Microbiology, Dasman Diabetes Institute, P.O. Box 1180, Kuwait City 15462, Kuwait; fatema.alrashed@dasmaninstitute.org; 6 Genetics and Bioinformatics Department, Dasman Diabetes Institute, Kuwait City 15462, Kuwait; alphonse.thangavel@dasmaninstitute.org; 7 Faculty of Medicine, Kuwait University, Kuwait City 13110, Kuwait

**Keywords:** FABP4, diabetes, obesity, BMI, FBG, HbA1c, ethnicity

## Abstract

Adipocyte P2 (aP2), also known as FABP4, is an adipokine that adipose tissue produces and expresses in macrophages. Its primary role is to facilitate the transportation of fatty acids across cell membranes. Numerous studies have reported associations between FABP4 and the development of metabolic disorders. However, there is limited knowledge regarding FABP4 expression in diabetes and obesity, especially about different age groups, genders, and ethnicities. This study aims to investigate the association between FABP4 levels, diabetes mellitus, and obesity within various ethnic groups. We measured plasma FABP4 concentrations in a cohort of 2083 patients from the KDEP study and gathered anthropometric data. Additionally, we collected and analyzed clinical, biochemical, and glycemic markers using multivariate regression analysis. The average FABP4 concentration was significantly higher in female participants than in males (18.8 ng/mL vs. 14.4 ng/mL, *p* < 0.001, respectively), and in those over 50 years old compared to those under 50 years of age (19.3 ng/mL vs. 16.2 ng/mL, *p* < 0.001, respectively). In this study, significant positive associations were found between the plasma level of FABP4 and obesity markers: BMI (r = 0.496, *p* < 0.001), hip circumference (r = 0.463, *p* < 0.001), and waist circumference (WC) (r = 0.436, *p* < 0.001). Similar observations were also seen with glycemic markers, which included HbA1c (r = 0.126, *p* < 0.001), fasting blood glucose (FBG) (r = 0.184, *p* < 0.001), fasting insulin (r = 0.326, *p* < 0.001), and HOMA-IR (r = 0.333, *p* < 0.001). Importantly, these associations remained significant even after adjusting for age, gender, and ethnicity. Furthermore, FABP4 levels were negatively associated with male gender (β: −3.85, 95% CI: −4.92, −2.77, *p* < 0.001), and positively associated with age (β: 0.14, 95% CI: 0.096, 0.183, *p* < 0.001), BMI (β: 0.74, 95% CI: 0.644, 0.836, *p* < 0.001), and fasting insulin (β: 0.115, 95% CI: 0.091, 0.138, *p* < 0.001). In this study, plasma FABP4 levels were significantly higher in diabetic and obese participants, and they were strongly influenced by age, gender, and ethnicity. These findings suggest that FABP4 may serve as a valuable prognostic and diagnostic marker for obesity and diabetes, particularly among female patients, individuals over 50 years old, and specific ethnic groups.

## 1. Introduction

Obesity and diabetes pose significant global health challenges, exacting a substantial toll on the world economy and contributing to a myriad of health issues worldwide [1]. Obesity is now ranked as the fifth most prevalent leading cause of death worldwide [2]. According to the World Health Organization (WHO), obesity is characterized by an “abnormal or excessive fat accumulation that may impair health” [3]. The fundamental cause of obesity lies in an energy imbalance between the calories consumed and those expended [4]. The multifaceted nature of obesity underscores its complex etiology, stemming from a dynamic interplay of genetic, environmental, and behavioral factors [5,6]. This complex interplay presents an alarming challenge for effective treatment, given the diverse array of genes and risk factors, including environmental and lifestyle elements, that contribute to the development of obesity [7]. While genetic predisposition plays a pivotal role in obesity, it is essential to recognize that genetic factors alone do not solely determine an individual’s susceptibility to obesity [8]. Rather, the interaction between genetic predisposition and environmental/lifestyle factors plays a critical role in weight regulation [9]. Therefore, attaining a comprehensive understanding of the underlying mechanisms driving obesity and weight gain is imperative. Lifestyle modifications, such as adopting healthier dietary habits and increasing physical activity, represent cornerstone interventions for managing obesity and diabetes. Nonetheless, the pressing need for innovative therapeutic approaches to address these pervasive health issues remains vital.

Fatty Acid Binding Protein 4 (FABP4), also known as adipocyte P2 (aP2), is an adipokine produced by adipose tissue and is also expressed in macrophages [1]. Its primary function is to facilitate the transportation of fatty acids across cell membranes [2]. Numerous studies have reported that FABP4 is associated with the development of metabolic disorders and cardiovascular diseases in conjunction with metabolic and inflammatory pathways [3,4]. Additionally, it has been shown to impact insulin sensitivity and thrombogenicity [5,6]. Xu et al., 2006, used a tandem mass-spectrometry-based proteomic analysis on 229 lean and obese participants to identify proteins secreted from adipocytes and present in human serum [10]. FABP levels are positively associated with blood pressure, dyslipidemia, waist circumference, fasting insulin, and the homeostasis model assessment of insulin resistance index [11,12]. Previous research has indicated that FABP4 may affect cardiomyocytes, contributing to coronary artery disease, heart failure, and diabetes mellitus [7,8,9]. Also, it has been shown that the serum FABP4 is associated with myocardial neutral lipid content in patients with type 2 diabetes mellitus (T2DM), where FABP4 has been found to induce insulin resistance by increasing the intracellular lipid contents [10]. Elevated levels of FABP4 have also shown a strong correlation with the deterioration of kidney function in diabetes patients [11,12]. Lee et al., 2019, assessed the baseline serum of FABP4 levels in 5454 Chinese participants from the Hong Kong West Diabetes Registry [13]. Circulating AFABP levels were predictive of the occurrence of adverse renal outcomes, even in participants with relatively well-maintained kidney function at baseline, suggesting its usefulness as a marker for early risk stratification in DKD [13]. On the other hand, FABP4 has been observed to augment insulin secretion by potentiating glucose-stimulated insulin secretion (GSIS) in both in vivo and in vitro studies [14,15]. This was also observed with glucagon, where FABP4 promotes hepatic glucose production irrespective of insulin, particularly in women with gestational diabetes mellitus (GDM) [14,16].

FABP4 is a typical intracellular adipokine responsible for enhancing lipid metabolism, storage, and, to some extent, transportation [15]. Excess fat cells in obese individuals lead to elevated levels of FABP4, which negatively affects various tissues, including the liver, pancreas, and heart [16]. Owing to the close relationship between excess body weight and cancer, several studies have reported an increased association between higher FABP4 levels and obesity-related cancer, particularly endometrial, ovarian, breast, and liver cancer, suggesting its therapeutic potential in cancer treatment [17]. FABP4 is also independently associated with elevated levels of triglyceride and reduced high-density lipoprotein (HDL), increasing the risk of heart disorders [18]. In type 1 diabetes mellitus (T1DM), FABP4 plays a regulatory role in glucose and lipid metabolism, particularly in ketogenesis during the insulin-deficient state [19]. Furthermore, a high level of FABP4 was also negatively associated with reversion from prediabetes to normal glucose tolerance (NGT) [20]. In recent years, many studies have proposed that FABP4 could serve as a valid biomarker for obesity, and early detection of diabetic nephropathy, T2DM, and GDM [19,21,22]. Moreover, FABP4 inhibitors have also been proposed as potential therapies for lipid disorders, particularly high triglycerides, acute kidney injury, obesity-related cancer, obesity, and diabetes [17,23,24,25].

Recognizing the pivotal role of FABP4 in metabolic disorders, it becomes crucial to investigate its relationship with variables such as age and ethnicity, particularly in regions where obesity and diabetes are prevalent. Therefore, the rationale for the current study is to investigate the association between FABP4, diabetes, and obesity and to explore the influence of age and ethnicity in a cohort from the Kuwait Diabetes Epidemiology Program (KDEP) study.

## 2. Results

### 2.1. Study Sample

The final sample size included in the analysis was n = 2083. Descriptive analysis indicated that the majority of patients were male (55.7%), with a median age of 45 years (min = 18, max = 82, IQR = 16), categorized as follows: 36% were less than 40 years old, 32.7% were between 40 and 50 years old, and 31.3% were over 50 years old. Additionally, 46.6% identified as being of Arab ethnicity, while 34.5% and 18.9% were South Asian and Southeast Asian, respectively. Moreover, the vast majority (69.2%) were non-diabetic, whereas around 30.8% had type 2 diabetes mellitus (T2DM). Over a third (40.2%) were overweight, and 38.7% were obese; in contrast, one-fifth (21.2%) were of normal weight. Furthermore, based on the HOMA-IR score, nearly half (49.7%) were insulin-resistant compared to 50.3% sensitive to insulin. The median hip circumference and waist circumference across the entire cohort was 102.3 cm and 95 cm, respectively. Furthermore, the median fasting glucose was 5.3 mmol/L, fasting insulin 7.9 mIU/L, and HbA1c 5.8%. More details are provided in Table 1.

**Figure 1:**
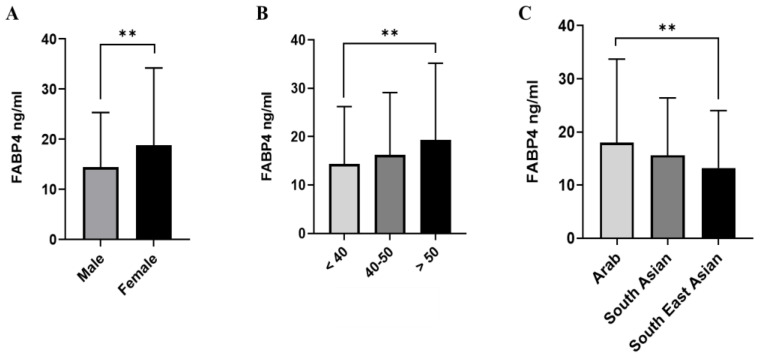
FABP4 level in plasma in all populations (n = 2083). The population was stratified based on gender (male and female) (**A**), age (>40, 40–50, and >50 years) (**B**), and ethnicity (Arab, South Asian, and Southeast Asian) (**C**). The level of FABP4 in plasma was determined using a multiplex bone panel. Statistical assessment was two-sided and considered statistically significant at ** *p* < 0.01.

**Figure 2:**
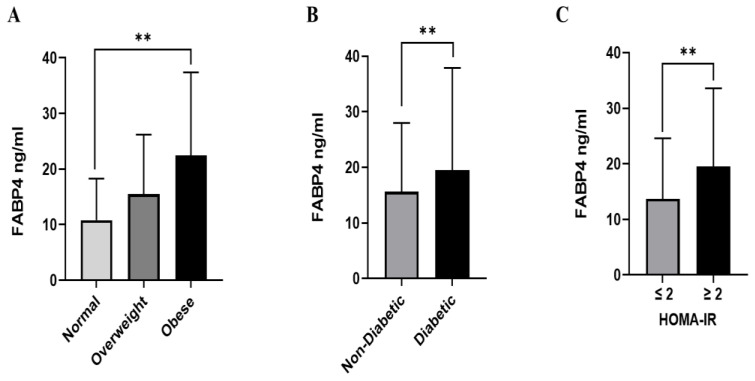
FABP4 level in plasma in all populations (n = 2083). The population was stratified based on BMI (BMI: >24.99 (normal), 25–29.9 (overweight), ≥30 (dese)) (**A**), diabetes status (non-diabetic and T2DM) (**B**), and insulin resistance (HOMA score: ≤2 (normal) and >2 (IR)) (**C**). The level of FABP4 in plasma was determined using a multiplex bone panel. Statistical assessment was two-sided and considered statistically significant at ** *p* < 0.01.

The average FABP4 concentration across the sample was significantly higher in the female participants than in the males (18.8 ng/mL vs. 14.4 ng/mL, *p* < 0.001, respectively, Figure 1A), and in those who were >50 years old than those <50 years of age (19.3 ng/mL vs. 16.2 ng/mL, *p* < 0.001, respectively, Figure 1B). Participants from Arab ethnic backgrounds had higher FABP4 levels than South Asian and Southeast Asian participants (18 vs. 15.6 and 13.2 ng/mL, *p* < 0.001, respectively, Figure 1C). Furthermore, obese individuals had a significantly higher level of FABP4 (22.5 ng/mL) compared to overweight (15.5 ng/mL) and normal weight individuals (10.8 ng/mL). Furthermore, patients with diabetes showed a higher level of FABP4 compared to those without diabetes (19.5 vs. 15.6 ng/mL, *p* < 0.001, respectively). Insulin-resistant patients had significantly higher FABP4 levels compared to those sensitive to insulin (19.5 vs. 13.7 ng/mL, *p* < 0.001, respectively) (see Table 2, Figure 2).

### 2.2. The Association between FABP4 Levels and Clinical Markers

In the multivariate analyses, it was observed that FABP4 levels exhibited statistically significant positive associations with several factors, including age (r = 0.228, *p* < 0.001), BMI (r = 0.496, *p* < 0.001), hip circumference (r = 0.463, *p* < 0.001), waist circumference (r = 0.436, *p* < 0.001), systolic blood pressure (SBP) (r = 0.079, *p* < 0.001), CRP (r = 0.316, *p* < 0.001), diastolic blood pressure (DBP) (r = 0.111, *p* < 0.001), fasting blood glucose (FBG) (r = 0.184, *p* < 0.001), HbA1c (r = 0.126, *p* < 0.001), fasting insulin (r = 0.326, *p* < 0.001), total cholesterol (TC) (r = 0.047, *p* < 0.001), total triglycerides (TG) (r = 0.159, *p* < 0.001), HOMA-IR (r = 0.333, *p* < 0.001), LDL (r = 0.022, *p* < 0.001), TSH (r = 0.088, *p* < 0.001), and FT4 (r = 0.087, *p* < 0.001), and a negative association with gender (r = −0.168, *p* < 0.001), HDL (r = −0.031, *p* < 0.001) and FT3 (r = −0.110, *p* < 0.001). Table 3.

### 2.3. The Association between FABP4 Levels and Markers for Obesity

In the current analyses, it was clear that the FABP4 levels demonstrated statistically significant positive associations with obesity markers including BMI (r = 0.496, *p* < 0.001), hip circumference (r = 0.463, *p* < 0.001), and waist circumference (WC) (r = 0.436, *p* < 0.001, Table 3).

### 2.4. The Association between FABP4 Levels and the Glycemic Indices

The analysis revealed a significant and positive association between FABP4 and HbA1c (r = 0.126, *p* < 0.001), fasting blood glucose (FBG) (r = 0.184, *p* < 0.001), fasting insulin (r = 0.326, *p* < 0.001), and the homeostatic model of insulin resistance (HOMA-IR) (r = 0.333, *p* < 0.001, Table 3).

In a secondary analysis using quantile median regression, and after adjusting for gender, age, and ethnicity, certain associations remained robust. Specifically, there was still a strong negative association between FABP4 levels and male gender (β: −3.85, 95% CI: −4.92, −2.77, *p* < 0.001), and South Asian ethnicity (β: −2.39, 95% CI: −3.88, −0.89, *p* < 0.002), and a positive association with age (β: 0.14, 95% CI: 0.096, 0.183, *p* < 0.001), BMI (β: 0.74, 95% CI: 0.644, 0.836, *p* < 0.001), TG (β: 1.9, 95% CI: 0.5–3.3, *p* < 0.007), and fasting insulin (β: 0.115, 95% CI: 0.091, 0.138, *p* < 0.001, Table 4).

## 3. Discussion

In the current study, we found that plasma FABP4 levels were significantly high among patients with diabetes and increased body weight. Additionally, FABP4 was positively and significantly associated with BMI, fasting insulin levels, and triglycerides (TG). These associations persisted after controlling for age, gender, and ethnicity. Our study demonstrates a significant association between FABP4 levels and increased body weight, suggesting that it could be a potential target for obesity treatment. In a study using an obese animal model, the effect of dual gene silencing of FABP4/5 targeted at white adipocytes using an adipocyte-targeting peptide resulted in significant weight loss, suppression of inflammation, and improvement in insulin sensitivity [15]. Additionally, in previously reported studies in both human and animal models, we observed a significant and positive association between high circulating levels of FABP4 and increased body mass, which aligns with our findings in the current study [17,18,19]. We also reported a significant association between FABP4 and increased insulin levels, a marker for insulin resistance. It has been suggested that FABP4 reduces insulin responsiveness by inhibiting peroxisome proliferator-activated receptor-Υ (PPAR-Υ), a crucial regulator of insulin response and adipogenesis [20]. Therefore, a recent study proposed that inhibiting FABP4 by administering the FABP4 inhibitor BMS309403 dramatically improves insulin sensitivity in obese mice [21]. In a cross-sectional study, the relationship between FABP4, insulin secretion, and insulin resistance in 12 T2DM patients and 18 controls was evaluated. After performing a hyperinsulinemic–euglycemic clamp, a strong negative relationship was found between the glucose disposal rate (GDR) and FABP4 and a positive correlation with insulin secretion and insulin sensitivity in T2DM patients [22]. It is important to acknowledge that insulin resistance is a risk factor for atherosclerosis. A previous study reported that high levels of FABP4 contribute to elevated blood pressure, which is another risk factor for atherosclerosis [23]. Similarly, in our study, we found that a high plasma level of FABP4 increases both systolic and diastolic blood pressure. On the other hand, we also found that FABP4 is associated with increased CRP levels. In a cross-sectional study that evaluated the circulating level of FABP4 in 43 morbidly obese and 38 lean women with no diabetes, there was a significant association between FABP4 and circulating CRP, HOMA-IR, and tumor necrosis factor (TNF) [24].

The thyroid hormone exerts a crucial role in maintaining carbohydrate and lipid metabolism, and its dysfunction facilitates the development of metabolic disorders [25]. Slight or modest elevations in thyroid hormones, including thyroid-stimulating hormone (TSH) and thyroxine (T4), have been reported in patients with obesity. In the current study, we observed a slight increase in TSH and T4, which is positively correlated with the high FABP4 levels [26]. Polak et al. recently reported, in a study of 66 females with polycystic ovary syndrome (PCOS) and 67 healthy controls, that serum concentrations of FABP4 were significantly higher in the PCOS group compared to the control group [27]. Another study, including an equal number of subclinical hypothyroid, overt hypothyroid, and healthy patients (n = 40 in each group), found that in patients with subclinical and overt hypothyroidism, the level of FABP4 was high, and the elevation closely correlated with high TSH [28].

Additionally, we observed significant racial differences in circulating FABP4 levels, especially among the South Asian cohort, highlighting the need for more inclusive studies and genetic exploration. Our study underscores the imperative for continued investigations aimed at uncovering the underlying mechanisms and genetic influences related to FABP4. These future studies are essential to fully establish the clinical utility of FABP4, paving the way for its potential applications in the realm of metabolic disorders. Recent data have demonstrated that FABP4 plays a role in mediating ferroptosis, a form of cell death characterized by iron-dependent fat accumulation, which has been linked to diabetic kidney disease and retinopathy. Additionally, the expansion of adipocytes, involving both an increase in number and size, contributes to adipose tissue hypertrophy. FABP4 has been shown to inhibit the FAT/CD36 signaling pathway, which is regulated by fatty acids during adipogenesis, through a negative feedback loop in adipocytes [29,30]. FABP4 also showed a strong association between aortic artery stiffness and heart failure [31]. In a cross-sectional study of 270 patients with diabetes and nondialysis chronic kidney disease, 92 patients showed aortic stiffness along with higher levels of FABP4, higher waist circumference and body fat mass [32]. Increased body weight and insulin resistance are correlated with a decline in fertility levels. Thus, data showed that in patients with polycystic ovary syndrome and endometriosis, there was an elevated level of FABP4, which is associated with reduced fertility [33]. Peripheral artery disease (PAD) is associated with amputation and mortality, particularly in patients with diabetes. However, there is no specific clinical biomarker for PAD. In a case–control study of 569 patients with PAD and 279 without PAD and followed-up for 3 years, patients who had higher FABP4 showed a significant association with worsening PAD status and vascular intervention [34]. Another study also showed a strong association between FABP4 and cardiovascular deaths in 12 years of follow-up. Surprisingly, in this study, even though female patients had higher levels of FABP4 than the male patients, a significantly higher rate of mortality was reported among the male participants [35]. However, a large multiethnic case–control study of postmenopausal women did not show any genetic variation in FABP4 that contributes to the pathogenesis of diabetes particularly in female patients [36]. There was also evidence that FABP4 has a significant influence on lipid metabolism by altering the classic lipid profile which is mediated by insulin resistance, particularly in patients with diabetes [37].

We must acknowledge several limitations in our study. The cross-sectional nature of our research restricts our ability to infer causative effects, suggesting that FABP4 may function as both a contributor to and a biomarker for diabetes and obesity. Genetic factors likely have a significant impact on circulating FABP4 levels, emphasizing the need for future investigations into different genetic variants that may play a role in FABP4 regulation.

Furthermore, our study represents a substantial stride in advancing our understanding of the potential significance of FABP4 in metabolic disorders, highlighting its promising role as a biomarker for cardiometabolic derangement. Future research and validation studies are crucial in elucidating the clinical significance of FABP4, both as a biomarker and as a therapeutic target for conditions such as diabetes and obesity, ultimately enhancing our ability to manage and address these pressing health concerns more effectively.

## 4. Methods and Materials

### 4.1. Participants and the Study Design

This is a cross-sectional analysis of blood samples from the Kuwait Diabetes Epidemiological Program (KDEP) study which was in Kuwait between 2011 and 2014. This study received approval from the Ethical Review Committee of Dasman Diabetes Institute (Protocol number RA2011-003) and was conducted according to the Declaration of Helsinki. All participants provided written informed consent before they participated in this study. A random sampling of the Kuwaiti population with proportional representation from each of the seven governorates was conducted for participant recruitment. A list of Kuwaiti residents, complete with their unique identification codes, was provided by the National Public Authority of Civil Information. A stratified random sampling technique was employed to select survey participants from this resident list. The survey design was adapted from the WHO STEPwise approach to surveillance (STEPS) methodology [38]. Individuals suffering from any infection and those aged younger than 21 were excluded. Recruitment took place at the Dasman Diabetes Institute between April 2011 and June 2014, with a dedicated team consisting of nurses, coordinators, interviewers, and phlebotomists.

### 4.2. Anthropometry and Vital Signs Measurements

Anthropometric measurements, including body weight, height, and waist circumference (WC), as well as vital signs such as blood pressure (BP), were recorded for each participant. BP was assessed using an Omron HEM-907XL digital sphygmomanometer (Kyoto, Japan). Three BP readings were taken with 5–10 min of rest between each reading, and the average values of the systolic and diastolic blood pressure readings were recorded. Height and weight were measured while the participants were dressed in lightweight indoor clothing and were barefoot, utilizing calibrated portable electronic weighing scales and portable inflexible height measuring bars. WC was determined using a constant tension tape at the conclusion of a normal exhalation, with the arms in a relaxed position at the sides, measured at the highest point of the iliac crest and the mid-axillary line. Body mass index (BMI) was calculated using the standard formula: body weight (in kilograms) divided by the square of height (in meters).

### 4.3. Laboratory Measurements

Blood samples were collected in Vacutainer EDTA tubes and used to measure clinical lab tests, as well as for long-term storage at −80 °C freezers until ready for assay. At the date of the visit, the collected blood was used to measure lipid and glycemic profiles using the Siemens Dimension RXL chemistry analyzer (Diamond Diagnostics, Holliston, MA, USA). The blood samples were used to measure lipid and glycemic profiles, including fasting plasma glucose (FPG), hemoglobin A1c (HbA1c), fasting insulin, triglycerides (TG), total cholesterol (TC), low-density lipoprotein (LDL), and high-density lipoprotein (HDL). HbA1c levels were measured using the VariantTM device (BioRad, Hercules, CA, USA). Insulin resistance was calculated using the homeostatic model assessment for insulin resistance (HOMA-IR) formula: (FBG in mmol/L) × (fasting insulin in mU/L)/22.5. The WHO criteria for the diagnosis of diabetes were used: FPG ≥ 7 mmol/L or HbA1c ≥ 6.5% (48 mmol/mol) [39]. Insulin levels were quantified using the Access Ultrasensitive Insulin Assay (Beckman Coulter, Brea, CA, USA), with both intra- and inter-assay coefficients of variation not exceeding 6%. HOMA-β was determined using the following formula: (20 × fasting insulin in mU/L)/(FBG in mmol/L − 3.5) multiplied by 100%.

### 4.4. FABP4 Plasma Levels and R&D Custom Multiplexing Assay

Plasma samples were extracted, aliquoted into plates, and stored at −80 °C for future use. For the multiplexing analysis, plasma samples were thawed and diluted 2× following the kit instructions for the Luminex custom-made panel (cat #LXSAHM, R&D, Minneapolis, MN, USA). The procedure was performed according to the kit instructions. In summary, plasma samples were diluted with the sample buffer provided in the kit. The kit standard was prepared with a 3-fold serial dilution. A cocktail of antibodies complexed with magnetic beads was diluted and aliquoted into a 96-well plate. The samples and standards were incubated in a diluted biotinylated antibody cocktail followed by a washing step and an incubation with diluted streptavidin-PE. Data were acquired using the Bioplex-200 (Bio-Rad, Hercules, CA, USA) using a 5-PL nonlinear standard curve setting in the Bio-Plex manager software version 6.0. No significant cross-reactivity with other proteins was observed. Intra-assay coefficients of variation ranged from 1.2% to 3.8%, whereas inter-assay coefficients of variation ranged from 6.8% to 10.2%.

### 4.5. Statistical Analysis

The statistical software STATA version 14 (STATA Corp., College Station, TX, USA) was used for data analysis. Initially, the data were examined for abnormalities and then recoded as necessary. Continuous variables were presented as the mean (SD) if the normality assumption was met; otherwise, the median (IQR) was reported. To assess significant differences between a continuous covariate dichotomized over a binary variable, the two-sample t-test was employed if the normality assumption for both groups was satisfied; otherwise, the Mann–Whitney U test was used. Differences in outcomes over a categorical exposure were evaluated using ANOVA if normality and homogeneity of variances were met; otherwise, the Kruskal–Wallis test was employed. To measure the strength of the correlation between binary and continuous variables, the point-biserial correlation coefficient was calculated. For two continuous covariates, the Pearson correlation coefficient was computed if normality was observed for both variables; otherwise, Spearman’s rank correlation coefficient was used. To model the relationship between a continuous outcome and a set of covariates in cases where the distribution of the outcome was skewed and outliers were present, quantile (median) regression was employed. This method is known for its robustness against outliers and its ability to handle overdispersion or underdispersion. In addition, the estimated robust variance–covariance matrix of the estimators (VCE) was obtained through bootstrapping. All statistical tests were two-tailed, and the significance level was set at 5%.

## 5. Conclusions

In summary, our study provides a comprehensive examination of the correlation between circulating FABP4 levels and obesity, diabetes, and the influence of age, gender, and ethnicity. We have demonstrated a significant positive association between FABP4 levels, fasting insulin levels, HbA1c, hip circumference, waist circumference, fasting blood glucose, HOMA-IR, and BMI. Additionally, we found a significant negative association between FABP4 levels and male gender. Importantly, age, gender, and ethnicity exerted a more substantial influence on FABP4 levels. These findings significantly enhance our understanding of FABP4’s potential role as a marker of metabolic health and its potential as a therapeutic target. Further research is needed to solidify our findings and enhance our understanding of FABP4 and its influence on age, gender, and ethnicity.

## Figures and Tables

**Table 1:** Demographic characteristics of 2083 participants.

Characteristics	(%) or Median (IQR)
Gender, n (%)	
Male	1161 (55.7%)
Female	922 (44.3%)
Age, n (%)	
<40	750 (36.0%)
40–50	680 (32.7%)
>50	653 (31.3%)
Ethnicity, n (%)	
Arab	899 (46.6%)
South Asian	666 (34.5%)
Southeast Asian	364 (18.9%)
Diabetes status, n (%)	
Non-Diabetic	1425 (69.2%)
Diabetic	633 (30.8%)
BMI, n (%)	
Normal BMI	441 (21.2%)
Overweight	837 (40.2%)
Obese	805 (38.6%)
HOMA-IR, n (%)	
HOMA-IR ≤ 2	969 (50.3%)
HOMA-IR > 2	958 (49.7%)
Hip circumference, median (IQR)	102.3 (13)
Waist circumference, median (IQR)	95 (15)
SBP, median (IQR)	131 (26)
DBP, median (IQR)	80 (16)
FBG, median (IQR)	5.3 (1.7)
Insulin, median (IQR)	7.9 (6.7)
TSH, median (IQR)	1.53 (1.14)
FT4, median (IQR)	11.78 (3.43)
FT3, median (IQR)	4.76 (0.78)
Age, median (min, max)	45 (18, 82)
HbA1c, median (IQR)	5.8 (1.2)
TC, median IQR)	5.1 (1.33)
AST, median (IQR)	21 (8)
CRP, median (IQR)	3 (2)

HOMA-IR: a homeostatic model of insulin resistance, BMI: is body mass index, IRQ: interquartile range, SBP: systolic blood pressure, DBP: diastolic blood pressure, FBG: fasting blood glucose, TSH: thyroid stimulating hormone, FT3: free triiodothyronine, FT4: free thyroxine, HbA1c: hemoglobin A1c, TC: total cholesterol, AST: aspartate transaminase, CRP: c-reactive protein.

**Table 2:** Descriptive analysis of FABP4 distribution across the 2083 participants.

Characteristics	Number of Participants	FABP4 Levels (ng/mL)	*p*-Value
Gender			
Male	924	14.42 (10.9)	<0.001 ^a^
Female	762	18.83 (15.4)	
Age			
<40	689	14.3 (11.9)	<0.001 ^b^
40–50	566	16.2 (12.9)	
>50	431	19.3 (15.9)	
Ethnicity			
Arab	715	18.0 (15.7)	<0.001 ^b^
South Asian	511	15.6 (10.8)	
Southeast Asian	310	13.2 (10.8)	
Diabetes Status			
Non-Diabetic	1404	15.6 (12.4)	<0.001 ^a^
Diabetic	261	19.5 (18.4)	
BMI			
Normal BMI	387	10.8 (7.5)	<0.001 ^b^
Overweight	691	15.5 (10.7)	
Obese	608	22.5 (14.9)	
HOMA-IR			
HOMA-IR ≤ 2	890	13.7 (10.9)	<0.001 ^a^
HOMA-IR > 2	644	19.5 (14.1)	

^a^ based on the Mann–Whitney U test, ^b^ based on the Kruskal–Wallis test, BMI: body mass index, HOMA-IR: a homeostatic model of insulin resistance, FABP4: fatty acid-binding protein 4.

**Table 3:** Correlations between FABP4 and the clinical, glycemic, and obesity markers in the 2083 patients.

Marker	FABP4 Level (r)	*p*-Value	Marker	FABP4 Level (r)	*p*-Value
Gender	−0.168	<0.001	HbA1c	0.126	<0.001
Nationality	0.144	<0.001	Insulin	0.326	<0.001
Age	0.228	<0.001	TC	0.047	<0.001
BMI	0.496	<0.001	TG	0.159	<0.001
Hip circumference	0.463	<0.001	HDL	−0.031	<0.001
Waist circumference	0.436	<0.001	LDL	0.022	<0.001
SBP	0.079	<0.001	TSH	0.088	<0.001
DBP	0.111	<0.001	FT4	0.087	<0.001
FBG	0.184	<0.001	FT3	−0.110	<0.001
CRP	0.316	<0.001	HOMA-IR	0.333	<0.001

CRP: c-reactive protein, FBG: fasting blood glucose, BMI: body mass index, DBP: diastolic blood pressure, SBP: systolic blood pressure, TSH: thyroid stimulating hormone, TC: total cholesterol.

**Table 4:** The adjusted analysis for the association between FABP4 and the clinical, glycemic, and obesity markers in the 2083 patients.

Marker	Unadjusted Median Regression β (95% CI)	*p*-Value	Adjusted Median Regression β (95% CI) *	*p*-Value
Male gender	−4.4 (−5.6, −3.2)	<0.001	−3.85 (−4.92, −2.77)	<0.001
Age	0.21 (0.15, 0.27)	<0.001	0.14 (0.096, 0.183)	<0.001
South Asian	−2.39 (−3.88, −0.89)	0.002	1.52 (0.488, 2.54)	0.004
BMI	0.915 (0.83, 1.0)	<0.001	0.74 (0.65, 0.84)	<0.001
Insulin	0.471 (0.27, 0.67)	<0.001	0.115 (0.091, 1.38)	<0.001
TG	1.9 (0.5, 3.3)	0.007	0.676 (0.067, 1.29)	0.030

* adjusted for age, ethnicity gender, and body mass index, the *p*-value was significant at <0.05. TG: triglyceride, BMI: body mass index, CI: confidence interval.

## Data Availability

The datasets used and/or analyzed during the study are available from the corresponding author upon reasonable request.
